# Comparison of Costs of Care for Medicare Patients Hospitalized in Teaching and Nonteaching Hospitals

**DOI:** 10.1001/jamanetworkopen.2019.5229

**Published:** 2019-06-07

**Authors:** Laura G. Burke, Dhruv Khullar, Jie Zheng, Austin B. Frakt, E. John Orav, Ashish K. Jha

**Affiliations:** 1 Department of Emergency Medicine, Beth Israel Deaconess Medical Center, Boston, Massachusetts; 2 Department of Health Policy and Management, Harvard T.H. Chan School of Public Health, Cambridge, Massachusetts; 3 Department of Medicine, Harvard T.H. Chan School of Public Health, Cambridge, Massachusetts; 4 Veterans Affairs Boston Healthcare System, Boston, Massachusetts; 5 Department of Health Law, Policy and Management, Boston University, Boston, Massachusetts; 6 Department of Biostatistics, Harvard T.H. Chan School of Public Health, Cambridge, Massachusetts; 7 Harvard Global Health Institute, Cambridge, Massachusetts; 8 Department of Healthcare Policy and Research, Weill Cornell Medical College, New York, New York

## Abstract

**Importance:**

Little empirical work has been performed on whether teaching hospitals are more expensive when considering total costs of care for an acute care episode.

**Objective:**

To compare total standardized costs at 30 days by hospital teaching status for common conditions.

**Design, Setting, and Participants:**

This cross-sectional study assessed the costs of hospitalizations among US Medicare beneficiaries 65 years and older at major, minor, and nonteaching hospitals from January 1, 2014, to November 30, 2015, for 15 medical conditions and 6 surgical procedures. Data analysis was performed from February 26, 2019, to April 16, 2019.

**Exposures:**

Hospital teaching status (major, minor, and nonteaching hospitals).

**Main Outcomes and Measures:**

The primary outcome was 30-day total standardized costs to Medicare for hospitalizations for all 21 conditions in aggregate as well as stratified by medical and surgical condition categories. Secondary outcomes included 30-day spending for individual components of care (index hospitalization, physician services, readmission, and outpatient and post–acute care services) as well as total standardized costs at 90 days.

**Results:**

The sample consisted of 1 249 006 hospitalizations at 3064 hospitals (232 [7.6%] major teaching, 837 [27.3%] minor teaching, and 1995 [65.1%] nonteaching hospitals). Treatment at a major teaching hospital was associated with lower total 30-day adjusted standardized costs ($18 605 vs $18 793 at minor teaching hospitals and $18 873 at nonteaching hospitals; difference between major and nonteaching hospitals: −$268; 95% CI, −$456 to −$80; *P* = .005). Treatment at a major teaching hospital was associated with higher spending for the index hospitalization ($8529 vs $8370 at minor teaching hospitals and $8180 at nonteaching hospitals; difference between major and nonteaching hospitals: $349; 95% CI, $308-$390; *P* < .001) but lower physician costs ($677 vs $725 at minor teaching hospitals and $728 at nonteaching hospitals; difference: −$50; 95% CI, −$60 to −$41; *P* < .001). Furthermore, post–acute care costs at 30 days were lowest at major teaching hospitals ($6015 vs $6239 for minor teaching hospitals and $6260 for nonteaching hospitals; difference: −$245; 95% CI, −$375 to −$115; *P* < .001). Thirty-day total costs were lower at major teaching hospitals compared with nonteaching hospitals for 12 of the 21 individual conditions examined. There was no difference in costs by teaching status at 90 days ($24 982 at major teaching hospitals vs $24 959 at minor teaching hospitals vs $25 044 at nonteaching hospitals; difference: −$61; 95% CI, −$310 to $188; *P* = .63).

**Conclusions and Relevance:**

Medicare patients treated at major teaching hospitals had lower Medicare spending at 30 days and similar costs at 90 days compared with Medicare patients at nonteaching hospitals. These findings appear to raise doubts that care at teaching hospitals is necessarily more expensive than care at nonteaching hospitals.

## Key Points

**Question:**

How does the cost of care vary by hospital teaching status for Medicare beneficiaries hospitalized for common medical conditions and surgical procedures?

**Findings:**

In this cross-sectional study of more than 1.2 million hospitalizations, major teaching hospitals had higher initial hospitalization costs than nonteaching hospitals, but the total costs of care at 30 days were lower at major teaching hospitals largely because of lower costs for post–acute care services and readmissions.

**Meaning:**

Total costs of care were similar or lower at teaching hospitals compared with nonteaching hospitals among Medicare beneficiaries treated for common medical and surgical conditions.

## Teaser

This cross-sectional study compares the 30-day costs of care for common conditions among US Medicare beneficiaries by hospital teaching status.

## Introduction

High and increasing health care costs have created a growing financial burden on patients and payers, leading to an effort to steer patients toward high-value institutions: those that deliver good outcomes at lower costs. Teaching hospitals, where health care professionals are educated and trained, are generally considered to be more expensive than nonteaching hospitals^1^ in part because of higher prices, and some insurers^2^ and policy makers^3^ have advocated shifting care from these institutions to lower health care spending for publicly and privately insured patients.

However, the degree to which treatment at major teaching hospitals is associated with higher health care spending in general and for Medicare, the largest and most consequential national payer, in particular, is not well understood.^4,5,6^ A few studies^4,5^ have focused on the initial hospitalization, at which spending is fixed by Medicare’s diagnosis related group (DRG) payment scheme. However, the particular site of hospitalization might have meaningful implications for total health care spending across the entire care episode if there are differences in spending on other services, such as post–acute care services, outpatient care, and readmissions. If these costs are higher at teaching hospitals compared with nonteaching hospitals, as is widely assumed, quantifying such costs is critical to understanding any tradeoff that might exist between cost of care and patient outcomes at teaching hospitals.^6,7,8,9,10^ Empirical evidence here would be helpful.

Given the paucity of recent comprehensive data on Medicare spending for comparable patients receiving care at teaching vs nonteaching hospitals and given the central importance of this issue as the Centers for Medicare & Medicaid Services (CMS) formulate policy that encourages patients to seek care from higher-value institutions, we sought to examine 3 questions. First, is hospital teaching status associated with differences in total spending for Medicare beneficiaries within 30 and 90 days of a hospitalization across a broad range of clinical conditions? Second, are there different patterns of spending on the individual components of cost associated with a hospitalization (eg, post–acute care services, outpatient care, and readmission) among major, minor, and nonteaching hospitals? Third, to what extent do these patterns of spending among teaching and nonteaching hospitals vary by clinical condition?

## Methods

### Study Design and Data Source

For this cross-sectional study, hospitalizations to acute care hospitals from January 1, 2014, to November 30, 2015, in all 50 states and the District of Columbia among continuously enrolled traditional Medicare beneficiaries, 65 years and older, were identified using the Medicare inpatient file. Analyses were limited to a random 20% sample identified by the CMS Research Data Assistance Center^11^ to identify all costs to Medicare within 30 and 90 days of the index hospitalization (most Medicare claims files are limited to the 20% sample). Hospital readmissions were identified in the inpatient file; claims for post–acute care services from the inpatient, skilled nursing facility, and home health claim files; physician claims from the carrier file; and outpatient claims from the outpatient file. Hospital characteristics were obtained from the Medicare Impact File, the American Hospital Association (AHA) annual survey, and the Medicare Cost Report. Critical access hospitals were excluded because they are not subject to the Inpatient Prospective Payment System,^12^ as were hospitals that lacked corresponding data in the AHA annual survey. Information on beneficiary characteristics (age, sex, race/ethnicity, and Medicaid eligibility) and death date was obtained from the Medicare Beneficiary Summary File and beneficiary chronic conditions from the Chronic Conditions Warehouse File. Race/ethnicity was determined by self-reporting and was included in the description of the study population by hospital teaching because differences in health care utilization and spending have the potential to contribute to health care disparities. The study followed the Strengthening the Reporting of Observational Studies in Epidemiology (STROBE) reporting guideline. The Office of Human Research Administration at the Harvard T.H. Chan School of Public Health approved this study. We did not obtain informed consent because the study data were obtained from previously collected, deidentified administrative Medicare claims data.

### Hospital Teaching Status

The primary determinant, hospital teaching status, was defined using 3 categories previously described in the literature.^7,9,13,14,15^ Major teaching hospitals were those that reported membership in the Council of Teaching Hospitals (COTH). Minor teaching hospitals did not have COTH affiliation but reported a medical school affiliation to the American Medical Association. All other hospitals were considered to be nonteaching hospitals. Additional hospital characteristics that we identified included the ratio of interns and residents to beds, size, geographic region, urban vs rural location, ownership status (public, for-profit, and private nonprofit), and intensive care unit availability. Each hospital was mapped to a hospital referral region (HRR) by zip code using the Dartmouth Atlas.

### Outcomes

The primary outcome was total standardized 30-day cost for each hospitalization among eligible beneficiaries. This method has previously been described by the CMS^16^ and used in health services research literature.^17,18,19,20^ Standardized cost is used to evaluate spending patterns nationally without considering regional differences in the price of services. Each service that is billed to Medicare is assigned the same cost based on national Medicare payment rates. First, the standardized cost of all fee-for-service Medicare claims at 30 days from the date of admission was determined and then divided by the following components previously described in the literature^17^: inpatient hospitalization, readmissions, post–acute care services, physician professional claims, and outpatient claims. Second, a posthospitalization cost after index category was created by aggregating all readmissions, post–acute care services, and outpatient costs. We did not include pharmacy claims because approximately 40% of beneficiaries do not have Medicare Part D coverage and there is no standardized fee schedule for pharmacy claims for calculating a standardized cost. Costs were identified for the 15 most common medical discharge diagnoses (acute myocardial infarction, pneumonia, congestive heart failure, arrhythmia, esophageal or gastric disease, chronic obstructive pulmonary disease, urinary tract infection, renal failure, gastrointestinal tract bleeding, hip fracture, metabolic disorder, respiratory tract disease, chest pain, stroke, and sepsis) and 6 common complex surgical procedures previously used to study costs and quality of surgical care (hip replacement, colectomy, coronary artery bypass grafting, endovascular abdominal aortic aneurysm repair, pulmonary lobectomy, and open abdominal aortic aneurysm repair).^17,21,22^ All costs were attributed to the hospital at which the index stay occurred regardless of the site of subsequent care (eg, readmission to a different hospital was attributed to the index hospital). The costs of care for hospitalizations ending in transfer (n = 20 710 [1.7%]) were assigned to the original hospital.

### Statistical Analysis

Analyses were conducted from February 26, 2019, to April 16, 2019. First, all hospitalizations among eligible beneficiaries to major, minor, and nonteaching hospitals were identified, as well as all subsequent fee-for-service claims to calculate 30-day standardized costs. The primary model was a generalized linear model assuming a γ distribution for costs and a log link. Generalized estimating equations were used to account for within-hospital correlation among patients. Estimated costs, with 95% CIs, for each hospital category were calculated using predictive margins (based on the online macro from SAS Institute Inc). The patient-level, 30-day total standardized cost was the outcome and hospital teaching status was the primary determinant. The model adjusted for HRR fixed effects (to account for regional differences in health policy and care delivery), as well as the principal discharge diagnosis (or procedure code for the 6 surgical conditions) and DRG weight to account for differences in the types of conditions and procedures treated at different institutions. A subsequent model also incorporated beneficiary age, sex, 27 chronic conditions, and Medicaid eligibility to adjust for differences in patient populations. These models were run for total standardized costs at 30 days for all hospitalizations across the 21 conditions in aggregate, medical conditions and surgical procedures, and individually for the 21 conditions. We also examined components of cost (eg, readmission spending). A γ model is no longer appropriate because many patients will not be readmitted and will therefore have zero cost. Instead, we assessed the differences in overall readmission costs (and other components of cost) among major teaching, minor teaching, and nonteaching hospitals using a linear regression model (with adjustments analogous to those described for the γ model). We then formalized the comparison among the 3 categories of hospitals using a zero-inflated, negative binomial model with the same covariate adjustments described above. This model allowed a comparison of costs among patients who were readmitted and had costs and, separately, a comparison of the rates of readmission among hospitals according to teaching status.

Because we focused on 3 primary comparisons between teaching and nonteaching hospitals (30-day adjusted total costs aggregated across all 21 conditions and for medical and surgical conditions), a 2-sided *P* < .02 was considered to be statistically significant. For comparisons of each of the 21 individual conditions, *P* < .002 indicated statistical significance. All other analyses were considered to be secondary.

### Sensitivity Analyses

#### Evaluation of 90-Day Costs

The main models were repeated for 90-day costs to determine whether the results were sensitive to longer periods of follow-up.

#### Examining Differences in Index Hospitalization Costs

To better understand any association between hospital teaching status and costs of the index hospitalization, we first ran a generalized linear regression model with a γ distribution for costs and a log link adjusting for DRG weight alone among all hospitalizations in the sample. Medicare allows for outlier payments for cases that incur extraordinarily high costs of care. To examine the degree to which differences in the proportion of these outlier cases accounted for any remaining differences in index hospitalization costs by hospital teaching status, we repeated the model with DRG weight only after excluding hospitalizations among any patients with outlier payments.

#### Inclusion of Indirect Medical Education Payments in the Cost Calculation

Indirect medical education (IME) payments are made by Medicare to teaching hospitals based on the ratio of interns and residents to beds to compensate teaching hospitals for the additional cost involving trainees in patient care. Although not considered to be health care spending in the traditional sense, IME payments nonetheless represent an additional cost to the Medicare program at teaching hospitals. Thus, we repeated our γ distribution models for 30-day standardized costs incorporating standardized IME payments to index hospitalizations and readmissions when taking these IME payments into account (eAppendix in the Supplement).

#### Propensity Weighting

To help ensure that comparisons between major teaching hospitals and nonteaching hospitals were adequately adjusted for patient severity, we used propensity weighting in addition to covariate adjustment. For each hospitalization, we used logistic regression to estimate the probability of treatment at a major teaching hospital based on principal diagnosis, HRR fixed effects, beneficiary age, sex, Medicaid eligibility, race/ethnicity, and hierarchical condition categories. We then incorporated the inverse of these probability weights in our γ regression models for total 30-day costs with hospital teaching status as the determinant (major vs nonteaching) and adjusting for the same set of covariates.

#### Other Models

We repeated our model for 30-day cost among a subset of hospitalizations in the lowest quartile of DRG weight. To account for any changes over time in market characteristics that may be associated with health care spending, we repeated our main model for total 30-day standardized cost incorporating yearly HRR-level Medicare advantage penetration and accountable care organization penetration (eAppendix in the Supplement). We also specified a model that incorporated other hospital structural characteristics (size, rural vs urban location, profit status, and post–acute care ownership). Finally, we examined the association between hospital teaching status and total 30-day standardized costs using the ratio of intern and residents to beds as a continuous determinant of teaching intensity using linear regression adjusting for DRG weight, condition or procedure, and patient characteristics. Analyses were conducted using SAS software, version 9.4 (SAS Institute Inc).

## Results

### Patient and Hospital Characteristics

This study assessed 1 249 006 hospitalizations among Medicare beneficiaries at 3064 hospitals. We excluded 6370 hospitalizations with missing data in the AHA annual survey and 17 843 for missing cost data. A total of 232 major teaching hospitals (7.6%) and 183 784 hospitalizations (14.7%) were represented, whereas 448 209 hospitalizations (35.9% of total) at 837 minor teaching hospitals were represented. A total of 1995 nonteaching hospitals (65.1%) and 617 013 hospitalizations (49.4%) were included in the sample. Other key differences in patient and hospital characteristics are reported in Table 1.

**Table 1.** Patient and Hospital Characteristics by Teaching Status^a^

Characteristic	Major Teaching	Minor Teaching	Nonteaching	Major Teaching and Nonteaching Standardized Difference
DRG weight, mean (SD)	1.85 (1.74)	1.66 (1.35)	1.54 (1.19)	0.12
Patient characteristics				
Total hospitalizations, No.	183 784	448 209	617 013	NA
Age, mean (SD), y	79.4 (8.7)	80.2 (8.6)	80.3 (8.6)	−0.10
Female	105 951 (57.6)	268 274 (59.9)	372 965 (60.4)	−0.06
Race/ethnicity				
White	148 700 (80.9)	388 167 (86.6)	547 320 (88.7)	−0.21
Black	25 571 (13.9)	38 353 (8.6)	43 844 (7.1)	0.22
Hispanic	2867 (1.6)	6755 (1.5)	9195 (1.5)	0.01
Other	4230 (2.3)	10 357 (2.3)	11 704 (1.9)	0.03
Medicaid eligible	36 682 (20.0)	86 579 (19.3)	135 329 (21.9)	−0.05
Comorbidity^b^				
Cancer	38 892 (21.2)	86 851 (19.4)	113 785 (18.4)	0.07
Diabetes	83 042 (45.2)	196 003 (43.7)	275 402 (44.6)	0.01
Renal failure	106 906 (58.2)	254 509 (56.8)	350 089 (56.7)	0.03
Ischemic heart disease	123 843 (67.4)	293 598 (65.5)	407 212 (66.0)	0.03
Mental illness	64 828 (35.3)	160 623 (35.8)	221 553 (35.9)	−0.01
Hospital characteristics				
Total hospitals	232 (7.6)	837 (27.3)	1995 (65.1)	NA
Size				
Small, <99 beds	1 (0.4)	104 (12.4)	824 (41.3)	−1.72
Medium, 100-399 beds	49 (21.1)	531 (63.4)	1107 (55.5)	−0.77
Large, ≥400 beds	182 (78.4)	202 (24.1)	64 (3.2)	1.79
Region				
Northeast	73 (31.5)	165 (19.7)	233 (11.7)	0.46
Midwest	57 (24.6)	242 (28.9)	408 (20.5)	0.09
South	72 (31.0)	265 (31.7)	969 (48.6)	−0.38
West	30 (12.9)	165 (19.7)	385 (19.3)	−0.17
Profit status				
For profit	8 (3.4)	120 (14.3)	507 (25.4)	−0.79
Nonprofit	172 (74.1)	636 (76.0)	1159 (58.1)	0.37
Public, nonfederal	52 (22.4)	81 (9.7)	329 (16.5)	0.16
With medical intensive care unit	218 (94.0)	714 (85.3)	1416 (71.0)	0.76
With cardiac intensive care unit	201 (86.6)	414 (49.5)	473 (23.7)	1.47
With post–acute care ownership	153 (65.9)	504 (60.2)	1005 (50.5)	0.32
HRR Medicare Advantage penetration	35 (15.1)	109 (13.0)	243 (12.2)	0.08
HRR-level ACO penetration^c^	78 (33.7)	284 (33.9)	642 (32.2)	0.03

Abbreviations: ACO, accountable care organization; DRG, diagnosis related group; HRR, hospital referral region; NA, not applicable.

^a^ Data are presented as number (percentage) unless otherwise indicated. Major teaching hospitals were members of the Council of Teaching Hospitals. Minor teaching hospitals had a medical school but no Council of Teaching Hospitals affiliation. Nonteaching hospitals had neither Council of Teaching Hospitals membership nor medical school affiliation.

^b^ Centers for Medicare & Medicaid Services Hierarchical Condition Categories.

^c^ Percentage of Medicare beneficiaries in the HRR who were attributed to an ACO.

### Total Adjusted Standardized 30-Day Costs by Hospital Teaching Status

**Figure 1.**
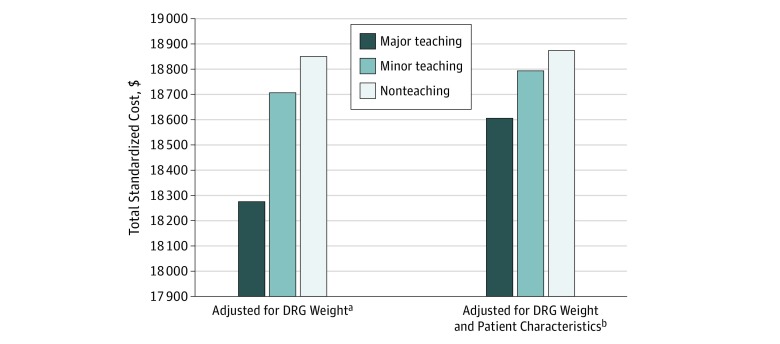
Total Standardized Costs Within 30 Days of a Hospital Admission by Hospital Teaching Status Total standardized costs for all hospitalizations for the 21 conditions in our sample (N = 1 249 006) are shown. The standardized cost method is described by the Centers for Medicare & Medicaid Services, in which each service is assigned a cost based on national Medicare payment rates without adjustments for wage index. Major teaching hospitals were members of the Council of Teaching Hospitals (COTH). Minor teaching hospitals had a medical school but no COTH affiliation. Nonteaching hospitals had neither COTH membership nor medical school affiliation. For the difference in costs between major and nonteaching hospitals in the model adjusted for diagnosis related group (DRG) weight, *P* < .001; in the model with patient characteristics added, *P* = .005. ^a^Generalized linear regression model with a γ distribution for costs and a log link, incorporating DRG weight, principal diagnosis or procedure, and fixed effects of hospital referral region. ^b^Model further incorporates patient age, sex, Medicaid eligibility, and chronic conditions.

Summary statistics for unadjusted standardized costs are presented in eTable 1 in the Supplement. When adjusting only for HRR fixed effects, diagnosis or procedure, and DRG weight, treatment at a major teaching hospital was associated with lower total standardized costs at 30 days for all hospitalizations in the sample ($18 275) followed by minor teaching ($18 706) and nonteaching hospitals ($18 850; difference between major and nonteaching hospitals, −$575; 95% CI, −$774 to −$376; *P* < .001) (Figure 1). After adjusting for patient characteristics, total 30-day standardized costs were $18 605 at major teaching hospitals, $18 793 at minor teaching hospitals, and $18 873 at nonteaching hospitals (difference between major and nonteaching hospitals, −$268; 95% CI, −$456 to −$80; *P* = .005). When examining medical conditions in aggregate, treatment at a major teaching hospital was associated with the lowest total standardized costs at 30 days ($18 375) compared with minor teaching ($18 561) and nonteaching hospitals ($18 634; difference between major and nonteaching hospitals, −$259; 95% CI, −$451 to −$67; *P* = .008) after adjusting for patient characteristics. For surgical procedures, treatment at a major teaching hospital was associated with lower total standardized costs, with $27 409 at 30 days compared with $27 839 at minor teaching hospitals and $27 960 at nonteaching hospitals (difference between major and nonteaching hospitals, −$550; 95% CI, −$940 to −$161; *P* = .006) (Table 2) in the model adjusting for patient characteristics.

**Table 2.** Comparison of 30-Day Adjusted Standardized Costs for Medical Conditions and Surgical Procedures by Service Type and Hospital Teaching Status^a^

Variable	Major Teaching	Minor Teaching	Nonteaching	Difference (95% CI)	*P* Value
**Medical Conditions (n = 1 171 792)**					
Total 30-day costs, $^b^	18 375	18 561	18 634	−259 (−451 to −67)	.008
Index hospitalization costs, $	8141	8081	8018	123 (99 to 147)	<.001
Physician costs, $	692	738	740	−48 (−58 to −38)	<.001
Cost after index hospitalization					
Odds ratio (95% CI)^c^	1.03 (1.01 to 1.04)	0.93 (0.92 to 0.94)	NA	NA	NA
Mean cost, $^d^	9010	9335	9384	−374 (−457 to −290)	<.001
Post–acute care services					
Odds ratio (95% CI)^c^	0.96 (0.95 to 0.98)	0.99 (0.99 to 1.00)	NA	NA	NA
Mean cost, $^d^	7142	7302	7257	−114 (−188 to −40)	.003
Readmission					
Odds ratio (95% CI)^c^	0.93 (0.92 to 0.94)	0.95 (0.94 to 0.96)	NA	NA	NA
Mean cost, $^d^	12 325	12 461	12 449	−123 (−228 to −18)	.02
Outpatient					
Odds ratio (95% CI)^c^	1.15 (1.14 to 1.16)	0.92 (0.91 to 0.93)	NA	NA	NA
Mean cost, $^d^	617	598	606	12 (5 to 19)	.001
**Surgical Procedures (n = 138 984)**					
Total 30-day costs, $	27 409	27 839	27 960	−550 (−940 to −161)	.006
Index hospital costs, $	14 263	14 165	14 181	82 (−50 to 215)	.22
Physician costs, $	598	675	666	−67 (−84 to −51)	<.001
Cost after index procedure					
Odds ratio (95% CI)^c^	1.00 (0.94 to 1.06)	0.88 (0.84 to 0.92)	NA	NA	NA
Mean cost, $^d^	10 109	10 565	10 695	−587 (−793 to −376)	<.001
Post–acute care services					
Odds ratio (95% CI)^c^	0.90 (0.85 to 0.94)	0.93 (0.89 to 0.96)	NA	NA	NA
Mean cost, $^d^	8437	8656	8731	−294 (−454 to −130)	<.001
Readmission					
Odds ratio (95% CI)^c^	0.94 (0.89 to 0.98)	1.02 (0.98 to 1.06)	NA	NA	NA
Mean cost, $^d^	13 156	13 553	13 710	−554 (−865 to −236)	<.001
Outpatient					
Odds ratio (95% CI)^c^	1.14 (1.10 to 1.18)	0.91 (0.88 to 0.94)	NA	NA	NA
Mean cost, $^d^	452	440	457	−5 (−19 to 9)	.47

Abbreviation: NA, not applicable.

^a^ The standardized cost method is described in the Outcomes subsection of the Methods section. Major teaching hospitals were members of the Council of Teaching Hospitals. Minor teaching hospitals had a medical school but no Council of Teaching Hospitals affiliation. The remaining hospitals were considered nonteaching.

^b^ Models for adjusted total 30-day standardized costs, index hospitalization, and physician costs were generalized linear regressions with a γ distribution for cost and a log link. Zero-inflated negative binomial regression models were specified to obtain adjusted costs for the following categories: cost after index, readmissions, outpatient care, and post–acute care services.

^c^ Odds ratio for having any use of the respective service type, with nonteaching hospitals as the reference group.

^d^ Mean costs among individuals who did have spending on the respective service type.

### Standardized 30-Day Costs by Spending Category and Hospital Teaching Status

**Figure 2.**
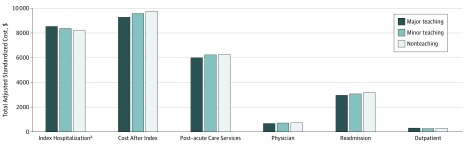
Total Adjusted Standardized Costs Within 30 Days of a Hospital Admission by Service Type and Hospital Teaching Status for All Conditions Generalized linear model with a γ distribution for costs and a log link adjusting for hospital referral region fixed effects, diagnosis related group weight, and patient age, sex, Medicaid eligibility, and chronic conditions. The standardized cost method is described by the Centers for Medicare & Medicaid Services, in which each service is assigned a cost based on national Medicare payment rates without adjustments for wage index. Major teaching hospitals were members of the Council of Teaching Hospitals (COTH). Minor teaching hospitals had a medical school but no COTH affiliation. Nonteaching hospitals had neither COTH membership nor medical school affiliation. ^a^Difference between major and nonteaching hospitals. All differences are significant at *P* < .001. Study sample includes 1 249 006 hospitalizations for the top 15 medical conditions and 6 surgical procedures.

When examining costs among hospitalizations for all 21 conditions in aggregate, treatment at a major teaching hospital was associated with the highest total spending at 30 days for the index hospitalization ($8529) followed by minor teaching ($8370) and nonteaching hospitals ($8180; difference between major and nonteaching hospitals, $349; 95% CI, $308-$390; *P* < .001) (Figure 2) as well as higher total spending on outpatient care (difference between major and nonteaching hospitals, $25; 95% CI, $14-$37; *P* < .001) after adjusting for patient characteristics. The opposite association was observed for readmission ($2961 at major teaching hospitals vs $3075 at minor teaching and $3205 at nonteaching hospitals; difference, −$244; 95% CI, −$348 to −$140; *P* < .001) (Figure 2), post–acute care spending ($6015 at major teaching hospitals vs $6239 at minor teaching and $6260 at nonteaching hospitals; difference, −$245; 95% CI, −$375 to −$115; *P* < .001), and physician costs ($677 at major teaching hospitals vs $725 at minor teaching and $728 at nonteaching hospitals; difference, −$50; 95% CI, −$60 to −$41; *P* < .001) (Figure 2). When comparing all postdischarge facility claims (post–acute care services, readmissions, and outpatient costs) by hospital teaching status, treatment at a major teaching hospital was associated with the lowest total spending ($9276 at 30 days compared with $9576 for minor teaching hospitals and $9743 for nonteaching hospitals; difference,−$467; 95% CI, −$467 to −$683; *P* < .001).

Using zero-inflated negative binomial models for medical conditions and surgical procedures (Table 2), we found that the lower total readmission spending seen at major teaching hospitals was associated with lower odds of having any readmission spending and lower mean spending per readmission event. For example, treatment at a major teaching hospital was associated with 7% lower odds of having any readmission spending (odds ratio, 0.93; 95% CI, 0.92-0.94; *P* < .001) for medical conditions and a difference in total readmission spending of −$123 (95% CI, −$228 to −$18; *P* = .02) among those with any readmission spending. This pattern was similar for readmission spending for surgical procedures and for post–acute care spending after hospitalizations for medical conditions and surgical procedures, whereas the association between hospital teaching status and outpatient use and spending was variable by condition category.

### Differences in 30-Day Costs by Hospital Teaching Status for Individual Conditions

**Figure 3.**
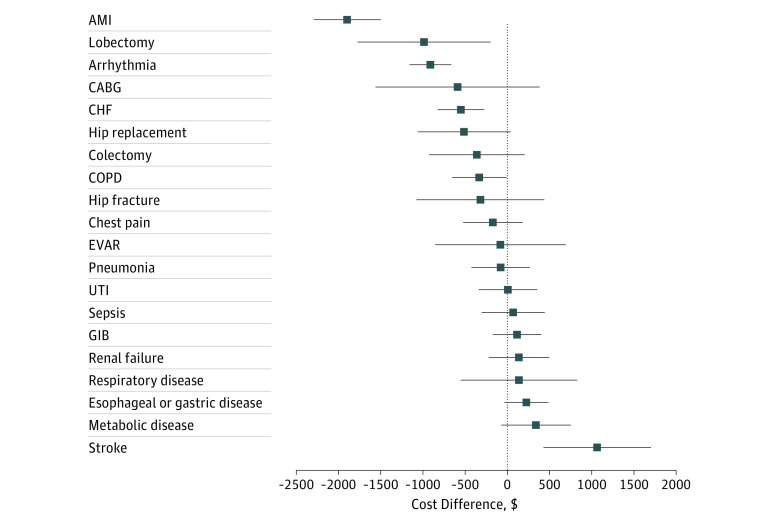
Comparison of the Difference Between Major Teaching and Nonteaching Hospitals With Respect to Adjusted Standardized 30-Day Costs for Individual Medical Conditions and Surgical Procedures Generalized linear models with a γ distribution for cost and a log link, adjusting for hospital referral region fixed effects, principal discharge diagnosis and/or surgical procedure, as well as diagnosis related group weight and patient age, sex, chronic conditions, and Medicaid eligibility. Major teaching hospitals were members of the Council of Teaching Hospitals. Minor teaching hospitals had a medical school but no Council of Teaching Hospitals affiliation. The remaining hospitals were considered nonteaching. Thestandardized cost method is described by the Centers for Medicare & Medicaid Services in which each service is assigned a cost based on national Medicare payment rates without adjustments for wage index. Displays costs based on 95% CIs. AMI indicates acute myocardial infarction; CABG, coronary artery bypass grafting; CHF, congestive heart failure; COPD, chronic obstructive pulmonary disease; EVAR, endovascular abdominal aortic aneurysm repair; GIB, gastrointestinal bleeding; and UTI, urinary tract infection.

Observed 30-day costs were lower at major teaching hospitals compared with nonteaching hospitals for 12 of 21 conditions (Figure 3). This association was statistically significant for 3 conditions (arrythmia: –$912 difference between major and nonteaching hospitals; 95% CI, –$1159 to –$665; *P* < .001; congestive heart failure: –$550 difference between major and nonteaching hospitals; 95% CI, –$825 to –$272; *P* < .001; and acute myocardial infarction: –$1897 difference between major and nonteaching hospitals; 95% CI, –$2295 to –$1501; *P* < .001. Treatment at a major teaching hospital was associated with higher total 30-day costs for patients hospitalized with stroke ($1064 difference; 95% CI, $428-$1701; *P* = .001). For medical conditions, a fairly consistent association was observed between treatment at a major teaching hospital and higher index hospitalization costs but lower spending after the index hospitalization (eTable 2 in the Supplement). There were no such associations for surgical procedures with the exception of coronary artery bypass grafting, for which teaching hospitals had higher index hospitalization costs ($20 289 at major teaching hospitals vs $19 509 at minor teaching hospitals vs $19 262 at nonteaching hospitals; difference between major and nonteaching hospitals, $1027; 95% CI, $574-$1481; *P* < .001). Small sample size for open abdominal aortic aneurysm repair precluded a determination of cost differences by hospital teaching status.

### Sensitivity Analyses

#### Total Standardized Costs at 90 Days by Hospital Teaching Status

After adjusting for patient characteristics, no association was found between hospital teaching status and total standardized costs at 90 days by hospital teaching status ($24 982 at major teaching hospitals vs $24 959 at minor teaching hospitals vs $25 044 at nonteaching hospitals; difference, −$61; 95% CI, −$310 to 188; *P* = .63) (eFigure 1 in the Supplement). A similar pattern was observed for medical conditions (eFigure 2 in the Supplement). However, for surgical procedures, treatment at a major teaching hospital was associated with lower total costs at 90 days ($32 557 vs $33 012 vs $33 265; difference, −$708; 95% CI, −$1172 to −$244; *P* = .003) (eFigure 2 in the Supplement).

#### Examining Differences in Index Hospitalization Costs

In our model, adjusting for DRG weight only, excluding hospitalizations among patients with outlier payments, reduced but did not eliminate the association between treatment at a major teaching hospital and higher index hospitalization costs (difference between major and nonteaching hospitals after excluding patients with outlier payments, $46; 95% CI, $25-$66; *P* < .001) (eTable 3 in the Supplement), suggesting that other add-on payments (eg, technology payment) may also play a role in driving these differences.

#### Accounting for IME Payments

When accounting for IME payments, index hospitalization costs were $10 062 at major teaching hospitals vs $8823 at minor teaching hospitals and $8535 at nonteaching hospitals (difference between major and nonteaching hospitals, $1527; 95% CI, $1406-$1648; *P* < .001) (eTable 4 in the Supplement). When considering total adjusted 30-day standardized costs, the adjusted costs were $1204 (95% CI, $955-$1454; *P* < .001) higher at major teaching hospitals.

#### Propensity Weighting

After incorporating propensity weighting in the model in addition to covariate adjustment, we found no association between treatment at a major teaching hospital and total 30-day standardized costs ($18 784 at major teaching hospitals vs $19 011 at nonteaching hospitals; difference, −$227; 95% CI, −$563 to $110; *P* = .18).

#### Other Models

The association between treatment at a major teaching hospital and lower 30-day standardized costs was again seen when restricting the analysis to hospitalizations among the lowest quartile of DRG weight and when further adjusting for yearly HRR accountable care organization and Medicare Advantage penetration (eTable 5 in the Supplement). No significant association was found between hospital teaching status and total 30-day standardized costs after adjusting for other hospital characteristics (difference between major and nonteaching hospitals, −$114; 95% CI, −$347 to −$119; *P* = .34) or when examining teaching intensity as a continuous determinant (coefficient for ratio of interns and residents to beds, $25 increase in total 30-day costs per each 0.1-unit change in hospital ratio of interns and residents to beds; 95% CI, −$14 to $64; *P* = .99) (eTable 6 in the Supplement).

## Discussion

We found that among Medicare beneficiaries hospitalized for common medical and surgical conditions, those hospitalized at a major teaching hospital had lower total standardized costs at 30 days compared with those at minor teaching hospitals and nonteaching hospitals. These differences persisted up to 90 days for surgical procedures, whereas costs of care were similar for medical conditions. Although initial hospitalization costs were higher at major teaching hospitals, in part owing to higher outlier payments for the sickest patients, spending on readmissions and post–acute care services was lower, leading to lower overall spending at 30 days. The lower readmission and post–acute care spending was associated with lower odds of having any readmission or post–acute care services use and lower mean costs among those who used these services. This pattern was largely consistent across individual medical and surgical conditions. When including IME payments, which are designed to pay for extra costs associated with training the next generation of physicians, major teaching hospitals had higher total spending at 30 and 90 days.

The association between treatment at a major teaching hospital and similar or lower total spending may seem unexpected given a general consensus that teaching hospitals are more expensive^1,23,24^ and that the involvement of trainees in patient care is relatively inefficient. This study suggests that although costs are somewhat higher for the initial hospitalization at major teaching hospitals, spending after hospital discharge, particularly on post–acute care services, is generally lower. The reason for this association is unclear. It is possible that greater treatment intensity^25^ or better care processes^5^ reduce downstream complications and the need for post–acute care services or that major teaching hospitals are able to coordinate more closely with post–acute care or other outpatient practitioners, thus reducing redundant or unnecessary care. Further study is needed to evaluate the mechanisms for lower postdischarge spending at teaching hospitals.

The finding of lower readmission costs for patients treated at teaching hospitals was surprising. Prior work^6,7,9,10^ suggests that mortality rates are lower at teaching hospitals; therefore, some of the sickest patients who might have died at other hospitals are likely surviving at teaching institutions. Thus, one would expect that their readmission costs would be higher. Furthermore, teaching hospitals have been penalized in the Hospital Readmission Reduction Program at higher rates than nonteaching institutions^26^ presumably because of higher rates of readmissions. The present study findings suggest that major teaching hospitals may have similar adjusted readmission rates and that the CMS may also be spending less in readmission costs for patients hospitalized at major teaching hospitals, presumably because patients are being admitted with lower-intensity DRGs.

These findings support efforts that might shift payments away from individual services toward clinical episodes across the entire care spectrum. Alternative payment models, including bundled payments, which encourage hospitals to take responsibility for spending beyond their traditional locus of control, are becoming more common.^27^ These results suggest that major teaching hospitals may be reasonably well positioned to adapt to these new payment models.

Whether IME payments should be included in total costs of care calculations is controversial. These payments were not included in the primary analysis because they were designed to help pay for the additional costs associated with teaching (eg, the potentially longer lengths of stay, additional testing). Inclusion of IME payments increased Medicare spending at teaching hospitals by approximately $1204 at 30 days. Whether those amounts are appropriate to support physician training is controversial but generally represent a different set of costs (that of training the next generation of physicians).

These findings are consistent with prior studies^4,5,6,17^ that found comparable costs for Medicare beneficiaries whether they received care at teaching or nonteaching hospitals. A 2012 study^5^ using Medicare cost reports found higher unadjusted costs per case at major teaching hospitals compared with minor and nonteaching hospitals but similar index hospitalization costs by hospital teaching status after adjusting for wage index, outlier costs, case mix, and patient socioeconomic factors. Another study^17^ of Medicare beneficiaries hospitalized for surgical procedures found that higher-quality hospitals had lower postdischarge spending and lower total costs to Medicare. Finally, our study is consistent with others^4,6^ that have found that medical education payments are associated with higher per-hospitalization Medicare spending. This study extended the prior work by examining total costs up to 30 and 90 days across a broad range of conditions.

### Limitations

Our study has a number of limitations. First, the analysis was limited to hospitalizations among Medicare beneficiaries 65 years and older, and the observed spending patterns may not be generalizable to other populations, particularly commercially insured patients, for whom prices for hospital services vary more widely.^28^ Second, this study did not consider how out-of-pocket spending for patients, an increasing concern for many beneficiaries, may differ by hospital teaching status. Third, because our study was observational, cost differences by hospital teaching status may be attributable to unmeasured factors, including differences in coding intensity. Fourth, Medicaid eligibility was used as a proxy for patient socioeconomic status, which may not capture broader social determinants of health that influence care delivery and costs.

## Conclusions

Treatment at a major teaching hospital was associated with lower costs at 30 days and similar costs at 90 days compared with treatment at nonteaching hospitals among Medicare beneficiaries hospitalized for common medical conditions and surgical procedures.

## Notes

Supplement. **eAppendix. **Additional Details on Cost Attribution and Index Hospitalization Standardization **eTable 1. **Distribution of Unadjusted Total 30-Day Standardized Costs by Hospital Teaching Status for Hospitalizations for Key Medical Conditions and Surgical Procedures **eTable 2. **Comparison of Adjusted 30-Day Standardized Costs for Selected Individual Medical Conditions and Surgical Procedures **eTable 3. **Comparison of Adjusted Costs for All Hospitalizations in Our Sample by Spending Component and Hospitals Teaching Status, Excluding Hospitalizations Among Patients Without Outlier Payments **eTable 4. **Comparison of Adjusted Standardized 30-Day Costs Including Indirect Medical Education (IME) Payments **eTable 5. **Thirty-Day Total Standardized Costs by Hospital Teaching Status and Adjustment Model **eTable 6. **The Association Between Hospital Teaching Intensity and Total Adjusted 30-Day Standardized Costs Using Intern/Resident: Bed Ratio (IRB) as a Continuous Predictor **eFigure 1. **Overall Standardized Costs Within 90 Days of a Hospitalization by Teaching Status and Adjustment Model **eFigure 2. **Total Adjusted Standardized Costs Within 30 and 90 Days of a Hospitalization for Selected Medical Conditions and Surgical Procedures to Major, Minor, and Nonteaching Hospitals
