# Errors in Author Affiliations

**DOI:** 10.1001/jamanetworkopen.2019.8316

**Published:** 2019-07-10

In the Original Investigation titled “Comparison of Costs of Care for Medicare Patients Hospitalized in Teaching and Nonteaching Hospitals,”^1^ published June 7, 2019, an affiliation was removed and another affiliation added for Dr Khullar and an affiliation was removed for Dr Jha. This article has been corrected.^1^
