# Supplementary material for: Comparison of Costs of Care for Medicare Patients Hospitalized in Teaching and Nonteaching Hospitals
Source: JAMA Netw Open. 2019 Jun 7;2(6):e195229. doi: 10.1001/jamanetworkopen.2019.5229 (PMC6563581; doi:10.1001/jamanetworkopen.2019.5229)
Supplement: Supplement. — eAppendix. Additional Details on Cost Attribution and Index Hospitalization Standardization eTable 1. Distribution of Unadjusted Total 30-Day Standardized Costs by Hospital Teaching Status for Hospitalizations for Key Medical Conditions and Surgical Procedures eTable 2. Comparison of Adjusted 30-Day Standardized Costs for Selected Individual Medical Conditions and Surgical Procedures eTable 3. Comparison of Adjusted Costs for All Hospitalizations in Our Sample by Spending Component and Hospitals Teaching Status, Excluding Hospitalizations Among Patients Without Outlier Payments eTable 4. Comparison of Adjusted Standardized 30-Day Costs Including Indirect Medical Education (IME) Payments eTable 5. Thirty-Day Total Standardized Costs by Hospital Teaching Status and Adjustment Model eTable 6. The Association Between Hospital Teaching Intensity and Total Adjusted 30-Day Standardized Costs Using Intern/Resident: Bed Ratio (IRB) as a Continuous Predictor eFigure 1. Overall Standardized Costs Within 90 Days of a Hospitalization by Teaching Status and Adjustment Model eFigure 2. Total Adjusted Standardized Costs Within 30 and 90 Days of a Hospitalization for Selected Medical Conditions and Surgical Procedures to Major, Minor, and Nonteaching Hospitals [file jamanetwopen-2-e195229-s001.pdf]

Supplementary Online Content

Burke LG, Khullar D, Zheng J, Frakt AB, Orav EJ, Jha AK. Comparison of costs of care for Medicare patients hospitalized in teaching and nonteaching hospitals. *JAMA Netw Open*. 2019;2(6):e195229. doi:10.1001/jamanetworkopen.2019.5229

eAppendix. Additional Details on Cost Attribution and Index Hospitalization Standardization

eTable 1. Distribution of Unadjusted Total 30-Day Standardized Costs by Hospital Teaching Status for Hospitalizations for Key Medical Conditions and Surgical Procedures

eTable 2. Comparison of Adjusted 30-Day Standardized Costs for Selected Individual Medical Conditions and Surgical Procedures

eTable 3. Comparison of Adjusted Costs for All Hospitalizations in Our Sample by Spending Component and Hospitals Teaching Status, Excluding Hospitalizations Among Patients Without Outlier Payments

eTable 4. Comparison of Adjusted Standardized 30-Day Costs Including Indirect Medical Education (IME) Payments

eTable 5. Thirty-Day Total Standardized Costs by Hospital Teaching Status and Adjustment Model

eTable 6. The Association Between Hospital Teaching Intensity and Total Adjusted 30-Day Standardized Costs Using Intern/Resident: Bed Ratio (IRB) as a Continuous Predictor

eFigure 1. Overall Standardized Costs Within 90 Days of a Hospitalization by Teaching Status and Adjustment Model

eFigure 2. Total Adjusted Standardized Costs Within 30 and 90 Days of a Hospitalization for Selected Medical Conditions and Surgical Procedures to Major, Minor, and Nonteaching Hospitals

This supplementary material has been provided by the authors to give readers additional information about their work.

eAppendix. Additional details of cost attribution

Repeat hospitalizations within 30 or 90 days were attributed to readmission costs for that time period. Any hospitalizations after that time period were considered another index hospital admission.

Calculation of standardized cost for the index hospitalization
We follow the Centers for Medicare and Medicaid Services methodology as described below. Details available at https://www.cms.gov/Research-Statistics-Data-and-Systems/Statistics-Trends-and-Reports/Medicare-Geographic-Variation/Downloads/Geo_Var_PUF_Technical_Supplement.pdf.

Standard allowed = (Labor base +Nonlabor Base + Capital Base) x DRG weight

+ [DRG Outlier Payment/ (Labor Ratio x Wage Index+ Nonlabor Ratio) + Capital Outlier Payment/Wage Index$^{0.6848}$] x Low Volume Adjustment, if applicable

+ New Tech Payment + Clotting Factor Payment

-Device Payment, if applicable

Calculating Indirect Medical Education (IME) Payments

There are two types of Indirect Medical Education (IME) Payments: Operating IME and Capital IME payments, which are calculated as follows:

Operating IME= ([Labor Base * wage index]+ Nonlabor Base) * DRG weight * 1.35 * [(1+IRB)$^{0.405}$-1]

Capital IME=([capital base] * DRG weight * e$^{0.2822\text{*residents/average daily census}}$

We included only Operating IME payments, which we calculated as follows:

Operating IME payment= Standardized Inpatient Payment *1.35 * [(1+IRB)$^{0.405}$-1]
The standardized inpatient payment corresponds to the following portion of the equation above but with wage index removed: (Labor Base+ Nonlabor Base)*DRG weight

We compared the difference between major and non-teaching hospitals using our standardized IME payment calculations and using actual IME payments from claims data, our results were similar.

© 2019 Burke LG et al. *JAMA Network Open.*

eTable 1. Distribution of unadjusted total 30-day standardized costs[a] by hospital teaching status[b] for hospitalizations for key medical conditions and surgical procedures[c]

	N Obs	Mean	Std Dev	25th pctl	Median	75th ptcl
**Major Teaching**	183784	$19,447	$18,132	$7,858	$13,409	$24,775
**Minor Teaching**	448209	$18,779	$17,207	$7,552	$12,869	$24,274
**Non-Teaching**	617013	$18,309	$17,140	$7,277	$12,417	$23,710

[a] Standardized cost methodology described by the Centers for Medicare and Medicaid Services in which each service is assigned a cost based on national Medicare payment rates, without adjustments for wage index. [b]Major teaching hospitals were members of Council of Teaching Hospitals (COTH). Minor teaching hospitals had a medical school but no COTH affiliation. The remaining hospitals were considered non-teaching. [c]The top fifteen medical discharge diagnoses and six common, complex procedures (colectomy, pulmonary lobectomy, coronary artery bypass grafting, endovascular abdominal aortic aneurysm repair, open abdominal aortic aneurysm repair and hip replacement).

© 2019 Burke LG et al. *JAMA Network Open.*

eTable 2. Comparison of adjusted 30-day standardized costs[a] for selected individual medical conditions[b] and surgical procedures[c].

Medical Conditions								Surgical Procedures						
		**Major Teaching**	**Minor Teaching**	**Non-Teaching**	**Difference (95% CI)**	**p**				**Major Teaching**	**Minor Teaching**	**Non-Teaching**	**Difference**	**p**
**Sepsis N=177,407**	**Index Hospitalization[d]**	$10,434	$10,333	$10,145	$289 ($244, $354)	<.001		**Hip replacement N=81,271**	**Index Hospitalization**	$12,212	$12,175	$12,271	-$59 (-$229, $110)	0.49
	**Cost after Index[e]**	$10,572	$10,812	$10,946	-$374 (-$819, $71)	0.10			**Cost after Index**	$15,208	$15,620	$15,613	-$406 (-$942, $130)	0.14
	**Total**	$21,757	$21,810	$21,688	$69 (-$304, $443)	0.72			**Total**	$28,227	$28,468	$28,739	-$513 (-$1,063, $38)	0.07
**CHF N=130,724**	**Index Hospitalization**	$7,381	$7,325	$7,267	$114 ($79, -$149)	<.001		**Colectomy N=26,013**	**Index Hospitalization**	$14,345	$14,499	$14,440	-$95 (-$332, $141)	0.43
	**Cost after Index**	$7836	$8175	$8511	-$675 (-$961, -$389)	<.001			**Cost after Index**	$9,425	$9.377	$9,247	$179 (-$627, $984)	0.66

© 2019 Burke LG et al. *JAMA Network Open.*

	**Total**	$15,926	$16,223	$16,475	-$550 (-$825, -$274)	<.001			**Total**	$24,010	$24,339	$24,371	-$361 (-$926, $205)	0.21
**Pneumonia N=102,273**	**Index Hospitalization**	$6,962	$6,970	$6,941	$21 (-$19, $62)	0.30		**Coronary Artery Bypass Grafting N=20,692**	**Index Hospitalization**	$20,289	$19,509	$19,262	$1,027 ($574, $1,481)	<.001
	**Cost after Index**	$7945	$7967	$8090	-$145 (-$525, $235)	0.46			**Cost after Index**	$9565	$11,512	$11,054	-$1,489 (-$2,564, -$413)	0.007
	**Total**	$15,650	$15,677	$15,729	-$80 (-$425, $266)	0.65			**Total**	$30,300	$31,573	$30,889	-$589 (-$1,562, $383)	0.24
**Arrhythmia N=98,369**	**Index Hospitalization**	$7,879	$7,829	$7,812	$67 ($31, $104)	<.001		**Endovascular Abdominal Aortic Aneurysm Repair N=5,859**	**Index Hospitalization**	$20,254	$20,277	$20,295	-$41 (-$181, $99)	0.57
	**Cost after Index**	$5279	$5762	$6009	-$730 (-$980,-$480)	<.001			**Cost after Index**	$4,883	$5,217	$4,896	-$13 (-$964, $939)	0.98
	**Total**	$13,740	$14,274	$14,652	-$912 (-$1,159, -$665)	<.001			**Total**	$25,627	$25,731	$25,709	$83 (-$856, $690)	0.83

© 2019 Burke LG et al. *JAMA Network Open.*

**Hip fracture**<br><br>**N=79,945**	**Index Hospitalization**	$12,418	$12,287	$12,304	$115<br><br>($42, $188)	0.002		**Lobectomy**<br><br>**N=4,500**	**Index Hospitalization**	$13,803	$13,861	$14,184	-$381<br><br>(-$769, $6)	0.054
	**Cost after Index**	$21,969	$22,484	$22,444	-$475<br>(-$1,238, $288)	0.22			**Cost after Index**	$4,695	$5,708	$5,332	-$637<br><br>(-$1,617, $343)	0.20
	**Total**	$35,248	$35,611	$35,567	-$320<br><br>(-$1,076, $437)	0.41			**Total**	$19,058	$19,797	$20,044	-$986<br><br>(-$1,774, -$198)	0.01

[a] Standardized cost methodology described by the Centers for Medicare and Medicaid Services in which each service is assigned a cost based on national Medicare payment rates, without adjustments for wage index. [b] Difference between major teaching and non-teaching hospitals. [c] Major teaching hospitals were members of Council of Teaching Hospitals (COTH). Minor Teaching Hospitals had a medical school but no COTH affiliation. The remaining hospitals were considered non-teaching. [d]Models for the outcomes of index hospitalization and total standardized costs were generalized linear regression with a gamma distribution for costs and a log link, adjusts for state fixed effects, principal discharge diagnosis and/or surgical procedure as well patient age, sex, chronic conditions and Medicaid eligibility. [e]Costs after index includes all outpatient, post-acute care, and readmission spending after the index hospitalization. For this outcome, a linear regression model with use with hospital teaching status as the primary predictor and adjusting for the same set of covariates.

© 2019 Burke LG et al. *JAMA Network Open.*

eTable 3. Comparison of adjusted[a] costs for all hospitalizations in our sample by spending component and hospital teaching status,[b] excluding hospitalizations among patients without outlier payments

Index hospitalization cost, All Hospitalizations	N	Major Teaching	Minor Teaching	Non-Teaching	Difference (95% CI)	p value
Adjusting for Diagnosis Related Group (DRG) weight, condition/procedure and patient characteristics	1,249,006	$8,515	$8,457	$8,391	$123 ($98, $149)	<.001
Adjusting for DRG weight only	1,249,006	$8,531	$8,480	$8,419	$113 ($87, $138)	<.0001
Adjusting for DRG weight only, outliers excluded[c]	1,221,720	$8,223	$8,222	$8,177	$46 ($25, $66)	<.0001

[a] Generalized linear model with a gamma distribution for costs with a log link incorporating Hospital Referral Region fixed effects, Diagnosis Related Group (DRG) weight, principal discharge diagnosis and/or surgical procedure as well as patient age, sex, Medicaid eligibility and chronic conditions. Standardized cost methodology described by the Centers for Medicare and Medicaid Services in which each service is assigned a cost based on national Medicare payment rates, without adjustments for wage index. [b]Major teaching hospitals were members of Council of Teaching Hospitals (COTH). Minor teaching hospitals had a medical school but no COTH affiliation. The remaining hospitals were considered non-teaching. [c] To examine the degree to which differences in proportion of these outlier case accounted for any remaining differences in index hospitalization costs by hospital teaching status, we repeated the model with DRG weight only after excluding hospitalizations among any patients with outlier payments.

© 2019 Burke LG et al. *JAMA Network Open.*

eTable 4. Comparison of adjusted[a] standardized 30-day costs including Indirect Medical Education (IME) Payments

	N	Major Teaching	Minor Teaching	Non-Teaching	Difference (95% CI)	P-Value
**Index Hospitalization, without IME**	1,249,006	$8,515	$8,457	$8,391	$123 ($98, $149)	<0.001
**Index Hospitalization, adding IME[b]**	1,242,020	$10,062	$8,823	$8,535	$1,527 ($1,406, $1,648)	<0.001
**Total Standardized Costs, without IME**	1,249,006	$18,605	$18,793	$18,873	-$268 (-$456, -$80)	0.005
**Total Standardized Cost with IME**	1,249,006	$20,174	$19,106	$18,969	$1,204 ($955, $1,454)	<0.001

[a] Generalized linear model with a gamma distribution for costs with a log link incorporating Hospital Referral Region fixed effects, Diagnosis Related Group (DRG) weight, principal discharge diagnosis and/or surgical procedure as well as patient age, sex, Medicaid eligibility and chronic conditions.
[b]Standardized IME payments (see eAppendix) were added to index hospitalization cost and for total standardized costs, readmission payments.

© 2019 Burke LG et al. *JAMA Network Open.*

eTable 5. Thirty-day total standardized costs[a] by hospital teaching status and adjustment model

		30-day Adjusted Costs for All Hospitalizations[b]				
**Cost Category**	**N**	**Major Teaching**	**Minor Teaching**	**Non-Teaching**	**Difference (95% CI)**	**P-value**
Hospitalizations in the lowest quartile of DRG weight [c]	301,786	$10,860	$11,109	$11,185	-$324 (-$491, -$158)	0.0001
Incorporating Time-varying market characteristics [d]	1,249,006	$18,605	$18,793	$18,874	-$269 (-$457, -$81)	0.005
Incorporating Hospital Characteristics [e]	1,248,625	$18,710	$18,813	$18,824	-$114 (-$347, $119)	0.34

[a] Standardized cost methodology described by the Centers for Medicare and Medicaid Services in which each service is assigned a cost based on national Medicare payment rates, without adjustments for wage index. [b]Hospitalizations for fifteen medical conditions and six surgical procedures(open abdominal aortic aneurysm repair, colectomy, pulmonary lobectomy, coronary artery bypass grafting, endovascular abdominal aortic aneurysm repair, and hip replacement). [c]Main model (generalized linear regression with a gamma distribution for costs and a log link) for 30-day mortality adjusting for HRR fixed effect, Diagnosis Related Group weight, condition/procedure and patient age, sex, Medicaid eligibility and chronic conditions was repeated restricting the analysis to hospitalization in the lowest quartile of DRG weight. [d]Main model (generalized linear regression with a gamma distribution for costs and a log link) for 30-day mortality was repeated for all hospitalizations in the sample, further adjusting for yearly HRR-level rates of Medicare Advantage penetration and percentage of total beneficiaries attributed to an Accountable Care Organization. [e]Main model (generalized linear regression with a gamma distribution for costs and a log link) for 30-day mortality was repeated for all hospitalizations in the sample, further incorporating additional hospital structural characteristics (hospital size, profit status, urban/rural location, and post-acute care ownership).

© 2019 Burke LG et al. *JAMA Network Open.*

eTable 6. The association between hospital teaching intensity and total adjusted 30-day standardized costs[a] using intern/resident: bed ratio (IRB) as a continuous predictor

	**Coefficient for IRB[b] (95% Confidence Interval)**	**p-value**
All Hospitalizations[c]	$25 (-$14, $64)	0.21
Medical[d]	$32 (-$8, $72)	0.12
Surgical[e]	-$31 (-$112, $50)	0.46

[a] Standardized cost methodology described by the Centers for Medicare and Medicaid Services in which each service is assigned a cost based on national Medicare payment rates, without adjustments for wage index. Adjustment model is a linear regression model with 30-day total standardized costs as the outcome and intern/resident to bed ratio as the predictor and incorporates fixed effects for hospital referral and incorporates principal diagnosis as well as patient age, sex, chronic conditions, Medicaid eligibility as covariates. [b] Change in total 30-day adjusted standardized costs per 0.1 unit increase in intern:resident to bed ratio. [c]All hospitalizations for the 21 conditions studied (N=1,249,006). [d]The top fifteen medical discharge diagnoses (N=1,171,792) and [e]six common, complex procedures (N=138,984**,** open abdominal aortic aneurysm repair, colectomy, pulmonary lobectomy, coronary artery bypass grafting, endovascular abdominal aortic aneurysm repair, and hip replacement). [c]

© 2019 Burke LG et al. *JAMA Network Open.*

eFigure 1. Overall Standardized Costs[a] within 90 Days of a Hospitalization by Teaching Status[b] and Adjustment Model

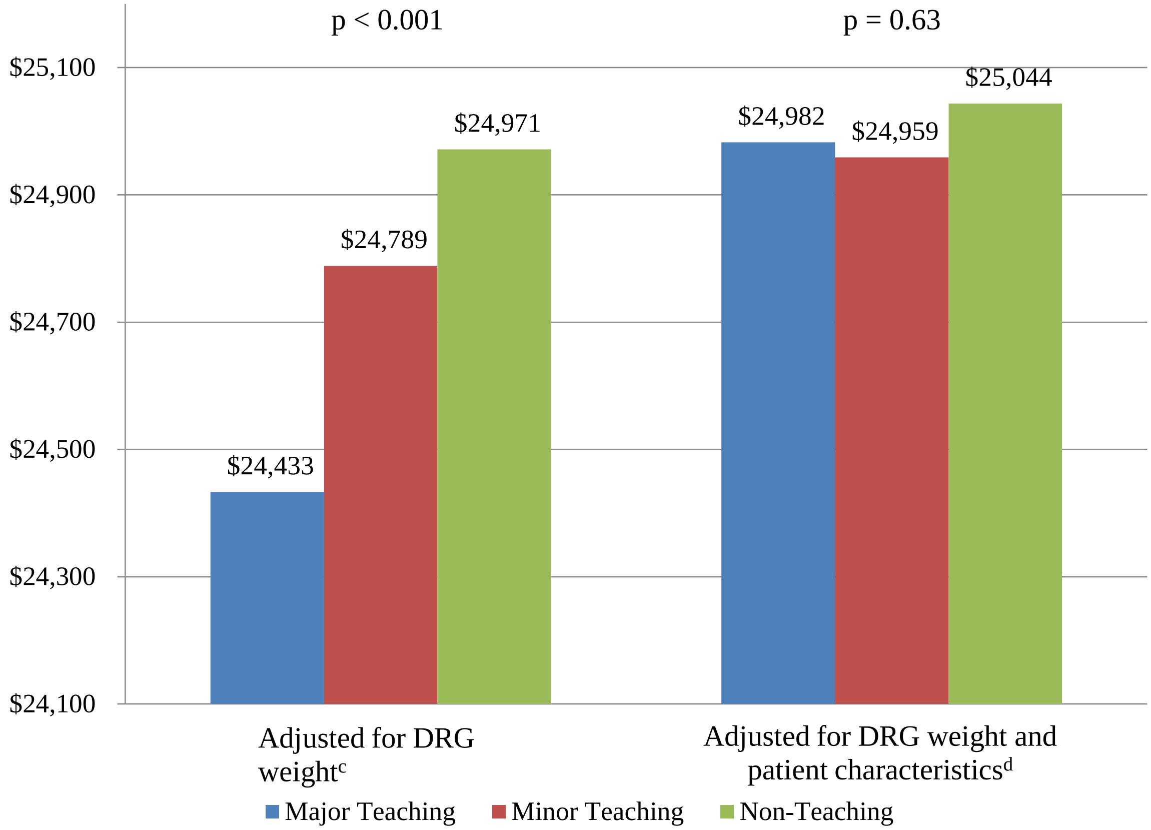

[a] Hospitalizations (N=1,249,006) for 15 key medical conditions and 6 surgical procedures among continuously-enrolled traditional Medicare beneficiaries from 2014-2015. Standardized cost methodology described by the Centers for Medicare and Medicaid Services in which each service is assigned a cost based on national Medicare payment rates, without adjustments for wage index. [b] Major teaching hospitals were members of Council of Teaching Hospitals (COTH). Minor teaching hospitals had a medical school but no COTH affiliation. The remaining hospitals were considered non-teaching. [c] Generalized linear model with a gamma distribution for costs with a log link incorporating Hospital Referral Region fixed effects, Diagnosis Related Group (DRG) weight, principal discharge diagnosis and/or surgical procedure. [d]Model further incorporates patient age, sex, Medicaid eligibility and chronic conditions.

© 2019 Burke LG et al. *JAMA Network Open.*

eFigure 2. Total Adjusted Standardized Costs[a] within 30 and 90 Days of a Hospitalization for Selected Medical Conditions and Surgical Procedures[b] to Major, Minor and Non-Teaching Hospitals[c]

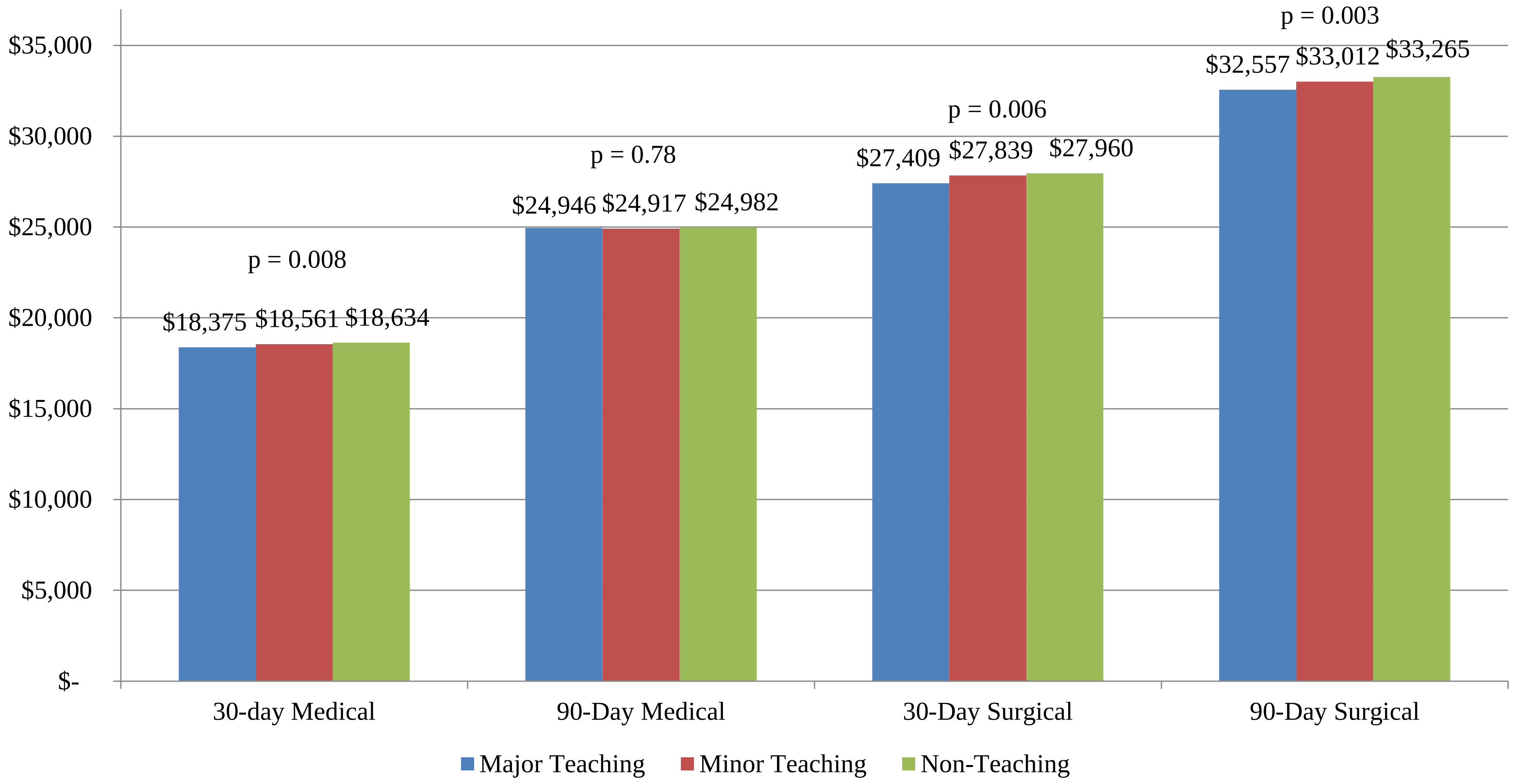

[a] Generalized linear model with a gamma distribution for costs with a log link incorporating Hospital Referral Region fixed effects, Diagnosis Related Group (DRG) weight, principal discharge diagnosis and/or surgical procedure as well as patient age, sex, Medicaid eligibility and chronic conditions. Standardized cost methodology described by the Centers for Medicare and Medicaid Services in which each service is assigned a cost based on national Medicare payment rates, without adjustments for wage index. Model incorporates state fixed effects, Diagnosis Related Group (DRG) weight, principal discharge diagnosis and/or surgical procedure as well as patient age, sex, Medicaid eligibility and chronic conditions. [b] The top fifteen medical discharge diagnoses (N= N=1,171,792**)** and six common, complex procedures (open abdominal aortic aneurysm repair, colectomy, pulmonary lobectomy, coronary artery bypass grafting, endovascular

© 2019 Burke LG et al. *JAMA Network Open.*

abdominal aortic aneurysm repair, and hip replacement; (N=138,984). [c] Major teaching hospitals were members of Council of Teaching Hospitals (COTH). Minor Teaching Hospitals had a medical school but no COTH affiliation. The remaining hospitals were considered non-teaching

© 2019 Burke LG et al. *JAMA Network Open.*
